# Molecular Analysis of A2-genes Encoding Stage-specific S Antigen-like Proteins among Isolates from Iranian Cutaneous and Visceral Leishmaniasis 

**Published:** 2011

**Authors:** Mahin Farahmand, Hasti Atashi Shirazi, Hossein Nahrevanian, Homa Hajjaran

**Affiliations:** 1 *Department of Parasitology, Pasteur Institute of Iran, Tehran, Iran*; 2 *Department of Parasitology, Institute of Public Health Research, Tehran University of Medical Sciences, Tehran, *

**Keywords:** A2protein, Iran, Leishmania, L .tropica, L .major, L. infantum

## Abstract

**Objective(s):**

Leishmania can lead to a broad spectrum of diseases, collectively known as leishmaniasis. The A2 gene/ protein family could be one of the most eligible candidate factors of virulence in visceral leishmaniasis (VL). The previous results confirmed that in *Leishmania* *infantum*, several A2 proteins are abundantly expressed by the amastigote, but not the promastigote stage. As there are no data available on the pattern of A2 gene / protein in Iranian Leishmania isolates of either cutaneous leishmaniasis (CL) or VL; the current study aimed to investigate molecular analysis of A2 gene in leishmania species among field isolates of .

**Materials and Methods:**

An A2 gene was identified by sequencing of crude PCR products resulting from 20 samples of CL and 10 samples of VL isolates from Iranian patients.

**Results:**

The results indicated the A2 gene in CL is only a single copy of 153 bp encoding a protein of 51 amino acids, as opposed to A2 of VL species with multi-copy genes of varying length. A2 sequences in Iranian* L. major* strains represented a homology with stage-specific S antigen-like protein (A2) of *L. major *and* L. infantum*. Moreover, A2 sequences in Iranian *L. tropica* strains have homology with A2 protein of *L. major* and *L. tropica*.

**Conclusion:**

It is concluded that A2 is an antigen candidate for vaccine development and diagnosis purposes and that A2 sequences are conserved among field isolates.

## Introduction

Protozoan parasites of the genus *Leishmania *are transmitted by the bite of infected sand fly to vertebrate hosts, leading to broad spectrum of diseases, collectively known as leishmaniasis. The clinical symptoms of the disease range from asymptomatic self-healing cutaneous lesions (CL), caused by *Leishmania* *major*, *L. tropica*, and *L. mexicana *species to mucocutaneous leishmaniasis (MCL) caused by *L. braziliensis* and severe visceral (VL) infections caused by *L. infantum*, *L. donovani*, and *L. chagasi* (1,2). Currently over 12 million people in 88 countries are infected with this parasite; 350 million are at risk of infection worldwide and 1.5-2 million new cases are reported every year (3). Both CL and VL are endemic in Iran (4,5), whereas anthroponotic *L. tropica* and zoonotic *L. major*, are observed with a high incidence rates in various parts of Iran (6,7). 

The diversity of clinical manifestation of leishmania infection depends on complex host-parasite relationships, where both the genetic or immunological status of the host and the proper parasite diversity in virulence appear as determinant factors (8,9). A number of parasitic factors have been identified to play a role in virulence/protection mechanisms in leishmaniasis (10). Since its first identification in *L. infantum *(11), several lines of evidence have indicated that the A2 gene/protein family could be one of the most eligible candidate factors of virulence in VL infections, and it is among the few widely accepted amastigote-specifics molecular markers identified to date (12-14). A2 genes were detected in *L. donovani*, *L. infantum and L. chagasi *(Old and New World VL) and in *L. mexicana *and* L. amazonensis *(New World DCL and MCL, respectively) and in CL species from the Old World (*L. tropica, L. aethiopica *and *L. major*) and the New World (*L. (V.) braziliensis, L. (V.) guyanensis *and *L. (V.) panamensis*) (15). 

Furthermore, intravenous injection of *L. major *genetically engineered to express A2, produced higher infection levels in the spleen than control *L. major*, further supporting the argument that A2 enhances survival in resident macrophages of visceral organs of mice (12, 14,16). A protective immunity can be achieved experimentally in mice by immunization with recombinant A2 protein or DNA vaccination which shows that A2 from *L. donovani *is highly immunogenic and represents a potential antigen for protection in VL (2,17). In addition, A2-antibodies were found in sera of human beings and dogs naturally infected with *L. chagasi* (18), and in patients with VL in Sudan and India and CL due to *L. mexicana*, while they were not detected in *L. tropica *and *L. (V.) braziliensis *infections (15). It has also recently shown that, A2 is not present in some leishmania species such as *L. tarentolae,* a non-pathogenic member of the genus *Leishmania *(1,19). 

In accordance to authors previous studies (20,21), other researchers confirmed that in *L. infantum*, as well as *L. donovani*, several A2 proteins are abundantly expressed by the amastigote, but not promastigote stage (16, 15,22). As there are no data available on the pattern of genes encoding A2-proteins in Iranian CL and VL isolates; this study was designed to investigate molecular analysis of this concept among field isolates of Iranian *Leishmania* species.

## Materials and Methods

*Cutaneous leishmania samples*

This study was carried on patients clinically suspected of CL, who were referred to the Department of Parasitology, Pasteur Institute of Iran, , for laboratory confirmation. The diagnosis of CL was based on clinical presentations of positive lesion smear and parasite culture. A checklist including personal, clinical and epidemiological data was completed for each case having cutaneous lesions before assay as described previously (21) (Table 1).

*Direct smear*

Samples for parasitological diagnosis were dermal scrapings of the active indurate margins of lesions or dermal scraping of the bottoms of the ulcers. Generally samples were obtained only from those sites which showed the most indurate margin. The lesion was cleaned of debris with saline solution, and debris was removed before sampling. Skin scrapings from the edge of the lesion were prepared; smears were stained with Geimsa and examined microscopically for the presence of amastigotes.

**Table 1.** Frequency of CL and VL collected samples by locations.

Endemic areas	CL patient samples	VL dog samples
Chabahar	2	0
Ghom	2	0
Kashan	3	0
Mashhad	1	0
Meshkin Shahr	2	0
Shiraz	3	0
Sabzevar	2	0
Torkamansahra	2	0
Varamin	3	0
Meshkin Shahr	0	10
Total number of samples	20	10

**Table 2.** Reference species used for comparison with samples.

Organism	Reference stock	Country of origin	Type of disease
*Leishmania major*	MRHO/IR/75/ER	Iran	CL
*Leishmania tropica*	MHOM/IR/09/Mash-F	Iran	CL
*Leishmania infantum*	MCAN/IR/96/Lon 49	Iran	VL

*Visceral leishmania sample*

Dogs are the principal mammalian hosts of* L. infantum*, which causes VL in the Mediterranean region and some areas of north western and southern Iran (23). Isolates of *L. infantum *were obtained from dogs of Meshkin Shahr district of Ardabil province, north western . Dogs in rural areas were selected randomly, and screened for* L. infantum* with their owners consent. The screening involved the collection of blood from the radial vein (5 ml/dog), the centrifugation of samples (800 g, 5 min) and the testing of sera (after incubation at 22 °C) for antileishmanial antibodies. Each serum was tested using a commercial rk39 dipstick (Cypress Diagnostics. Langdorp, Belgium) and a direct agglutination test (DAT) (kindly provided by the Protozoology Unit, Department of Parasitology, Institute of Public Health Research, Tehran University of Medical Sciences, Tehran, Iran), based on* L. infantum *antigens with a cut-off titer for DAT positivity of 1:320 (24). With the approval of the local health authority, the seropositive dogs were killed by terminal anesthesia, transferred to the Parasitology Laboratory at Meshkin Shahr Health Research Centre, and then carefully dissected so that parasites could be isolated in culture.

*Culture method*

CL samples collected from skin lesions of patients or spleen and liver of VL infected dogs, were cultured in both liquid phase of Novy- Macneal- Nicole (NNN) and in RPMI-1640 media (Sigma) (pH 7.2), supplemented with 20% fetal calf serum (Invitrogen, UK) and 2 mM L-glutamine, 55 µg/ml penicillin, and 125 µg/ml streptomycin. The culture was incubated at 25 °C and checked for growth of *Leishmania* promastigotes and checked every day using an inverted microscope for 28 days as described previously (21, 22,25).

*Parasites*

Samples including 10 strains of visceral (*L. infantum*) and 20 strains of Old World cutaneous *Leishmania* (*L. (L.) major, L. (L.) tropica*) were used for sequencing. Reference strains of above leishmania parasites as indicated in Table 2were also used for comparison and accuracy of assay. 

*DNA extraction*

Parasites were washed three times with PBS and DNA extraction was performed using QIA Amp DNA Mini kit () according to the manufacturer manuals (200 µl PBS / 10^6^ parasites).

*Detection of species*

**Figure 1.**
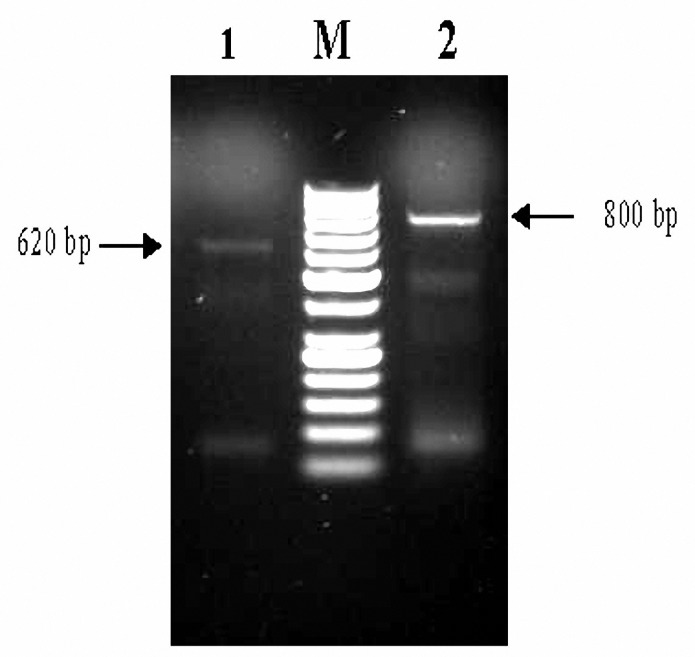
PCR detection of all leishmania parasites from CL samples.  1 *L. major, *2* L. tropica, *M 50-bp DNA ladder

All isolates were detected according to the kit manufacturer using *Leishmania *sp*.* PCR Determination kit (Cinnagen Company, Iran, www.cinnagen.com) (5,26). This kit is designed for qualitative detection of *Leishmania *sp*.* kinetoplast DNA by PCR. A single 620 bp band for identifying of *L. major* and a 800 bp band for detection of *L. tropica* were evidenced (Figure 1).

*PCR*

Amplification of the parasite DNA matrix (50 ng) was made using L2/R3 primers (5'-GGCAATGCGAGCGTCACAGTC/ 5'- CAACGCGTACGATAATGCCACA). The L2/R3 primers were applied according to Garin *et al* 2005 (2). The PCR was performed in a reaction mixture of 50 µl containing either 1 or 3 mM MgCl_2_, 200 M each dNTP, 5 pmol of each primer (TEB Technology Ltd, Tehran, Iran), 1U Taq polymerase (Roche, Germany). L2/R3-PCR conditions consisted to denaturation at 94 °C for 3 min, followed by 35 amplification cycles at 94 °C for 1 min, 58 °C for 1 min, 72 °C for 1 min, then one cycle at 72 °C for 5 min. Five µl of PCR product was electrophoresed in 2% agarose gel in the presence of ethidium bromide, and visualized under UV Light. A 50-bp DNA ladder (Fermentase, ) was used as DNA marker.

*Sequencing*

For sequencing, the two strands of PCR-amplified DNA were purified with QIA quick 

PCR Purification Kit (Qiagen,USA) and sequenced with the corresponding PCR primer set using Dideoxy chain termination procedure (Chemistry V3.1, Applied Biosystems) and the 3730XL DNA analyzer (Applied Biosystem) by Millen Gene sequencing Service (Labège, France).

*Gene bank accession numbers*

NCBI accession numbers for genomic DNA and putative protein sequences are GU235991, GI: 281490070 for *L. major*, GU376735 and GI: 288551599 for *L. tropica*.

## Results

**Figure 2.**
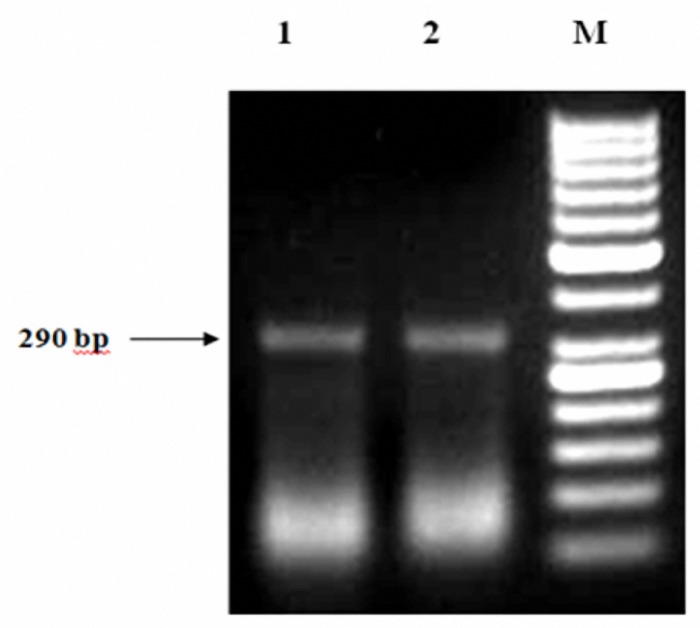
Comparison of PCR results from *L. major* between standard and isolates from CL patients. 1 *L. major* MRHO/IR/75/ER, 2 *L. major *isolates from CL patient, M 50-bp DNA ladder, Arrow A2 gene

**Figure 3:**
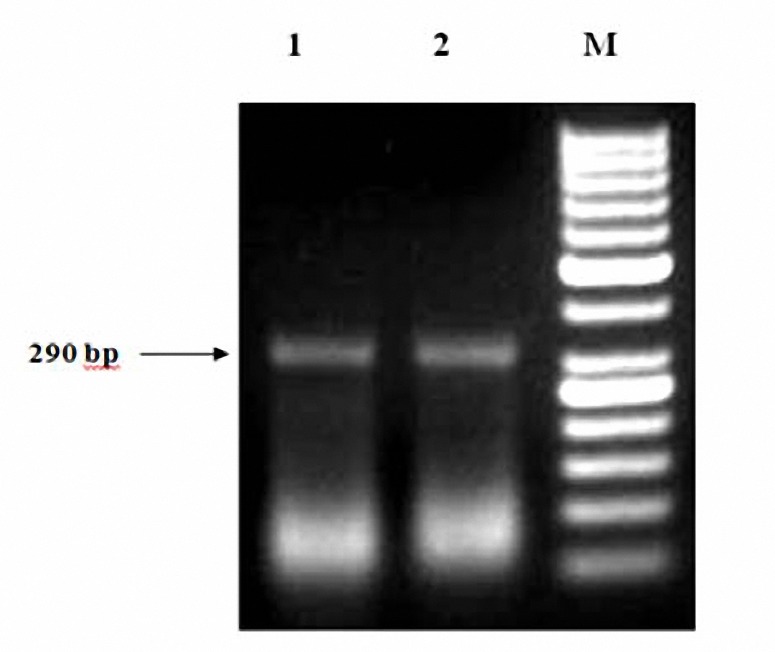
Comparison of PCR results from *L. infantum *between reference and isolates from VL dog samples. 1 *L. infantum* MCAN/IR/96/Lon492, 2* L. infantum *isolate from dog samples, M 50-bp DNA ladder, Square Bracket A2 gene area

In current study, L2/R3 primers encoding crude genomic DNA of parasite was used for differentiation of leishmania species by unique electrophoretic pattern.  For entire CL isolates, one single band of approximately 290 bp (1-3 mM MgCl_2_) was detected, while in VL isolates, two approximate sizes of 320 and 550 bp bands (3 mM MgCl_2_) were identified (Figures 2,3).

The products in VL species were shown by direct sequencing, which may be a mixture of A2 sequences and probably non-specific products. A single sequence of 290 nucleotides was evidenced by direct sequencing of the crude L2/R3 PCR products of the genomic DNA from all *L. major *and *L. tropica *strains. Seventeen strains of *L. major* among Iranian samples were detected, which were identical and comparable with IPAP/MA/86/ LEM898 and MHOM/SU/73/5 ASKHLEM134 except for a single polymorphism of C/G at position 155. Three strains of *L. tropica *were observed, which were identical with *L.* *tropica* AY255806 except for a single polymorphism G/A at position 35. Two A2-gene sequences of 320 and 550 nucleotides were isolated from the genomic DNA of *L. infantum*. These sequences were comparable with *L. infantum* AY255809 and AY255807 and *L. major *MRHO/IR/75/ER and *L. tropica* MHOM/IR/09/Mash-F.

## Discussion

In the present study, a single sequence of 290 nucleotides was obtained by direct sequencing of crude DNA products from all CL *Leishmania.* The amplification of Old World *Leishmania *genomic DNA with L2/R3 A2-gene primer set resolved in a single amplification product on gel electrophoresis, as opposed to the complex pattern observed with *L. infantum*. 

A2 sequences were identified in three samples of *L. tropica *strain isolated from CL patients. The sequences were 88% (207nt/236) identical to the *L. major* strain MRHO/IR/75/ER, S antigen-like protein (A2) gene (PubMed: GU235991.1) and *L. tropica* A2-gene, A2-type I allele (PubMed: AY255806.1). In addition, A2 sequences were identified in 16 strains of *L. major *from CL patients and MRHO/IR/75/ER strain, respectively. These sequences were 88% (210nt/236) identical to *L. major* strain MHOM/SU/73/5 ASKH LEM134 S antigen (PubMed: AY185122.1) and 89% to *L. major *strain MHOM/IR/-/173 A2-gene (PubMed: AF532103.1), and 95% to *L. infantum* homolog A2- mRNA (PubMed: S69693.1).

The identified A2 gene family in *L. donovani* is reported to be specific to the amastigote stage, determining visceral infections (11,14). A2 genes were also shown to be organized in several clusters each comprising multiple A2 genes of varying length that are tandemly associated with related sequences (27). In the previous studies, three different A2-allele types II, III and IV were sequenced from the* L. infantum *MHOM/FR/92/ LEM2385 Clone-1 genomic library. This provided additional evidence that A2 of VL species is a multiple gene family. Types I, II and III alleles differ only in the number and arrangement of the repeated motives at the 3' end variable region of the gene (2,11). In the study which was conducted by Garin *et al *2005 (2), they identified A2 sequences showing a limited number of repeats and consequently a length of only 371-464 bp contrasting with the previously published A2 genes of about 700-800 bp. The A2 sequence shared by all strains and species of *Leishmania *presented a single polymorphism C/G at position 155 for *L. major* and G/A at position 35 for *L.* *tropica* strains. These results are compatible with Garin *et al*, 2005 (2), who indicated that the A2 gene of Old World CL was highly conserved. In VL isolates the multiple sequences of varying length have been observed, which may be associated with multiple parasite populations in naturally infected hosts. 

## Conclusion

It can be concluded that A2 sequences in* L. major *strains has homology with A2-genes encoding stage-specific S antigen-like proteins of *L. major *and* L. infantum*. A2 sequences in *L. tropica *strains have also represented a homology with A2-gene encoding proteins of *L. major *and* L. tropica*.
